# Perspectives of clinicians caring for people experiencing homelessness on point of care tests: an online survey

**DOI:** 10.1136/bmjopen-2025-113834

**Published:** 2026-07-16

**Authors:** Frances Rose, Umasha Ukwatte, Neil Singh, Philip J Turner, Merlin Willcox, Belinda Lennox, Gail N Hayward

**Affiliations:** 1 Nuffield Department of Primary Care Health Sciences, University of Oxford, Oxford, UK; 2 Department of Primary Care and Public Health (PCPH), Brighton and Sussex Medical School, Brighton, UK; 3 Primary Care and Population Sciences, University of Southampton Faculty of Medicine, Southampton, UK; 4 Department of Psychiatry, University of Oxford, Oxford, UK

**Keywords:** homelessness, point of care systems, primary health care, technology

## Abstract

**Abstract:**

**Objectives:**

People experiencing homelessness (PEH) face multiple barriers to seeking healthcare and have poorer health outcomes. Point of care tests (POCTs) provide a potential solution to improving access to diagnostics for this population. This survey aimed to understand if these technologies could address unmet needs in primary care for PEH.

**Design and setting:**

An online survey was circulated via dedicated inclusion healthcare newsletters to professionals providing community-based care for PEH in England. The survey focused on experiences of diagnostics and opinions on the use of POCTs for this population.

**Participants:**

Thirty-two healthcare workers participated, including GPs, nurses and other allied practitioners from 13 different Integrated Care Boards across England.

**Analysis:**

Descriptive analyses were performed using standard statistical parameters. A reflexive thematic analysis was performed on free-text responses.

**Results:**

There was evidence of current POCT use but with marked variation across services as to which tests are available. Healthcare workers were overwhelmingly positive about the potential for POCTs, with rapid results facilitating prompt diagnosis and management, increasing likelihood of engagement. C reactive protein testing was considered as the test, which could confer the most benefit to acute care, with renal function and troponin also being discussed, whereas tests to determine cardiometabolic risk were thought to have the most patient benefit in chronic care. Point of care ultrasound for diagnosis of respiratory pathologies and deep vein thrombosis and POCTs for malnutrition were suggested as potential future technologies to address unmet healthcare needs.

**Conclusion:**

The majority of responders expressed that enhancing the provision of POCTs would be beneficial, both in acute and chronic care scenarios, due to the benefits of getting rapid results and reducing the need for repeat appointments or onward referral for diagnostics.

**STRENGTHS AND LIMITATIONS OF THIS STUDY**

- A strength of this survey is that it considers affordable and practical solutions, in the form of point of care tests (POCTs), to address significant unmet health needs for this underserved population.
- The methodology used allowed collation of responses from across England, from multiple different primary healthcare delivery services, but did not allow as much exploration of issues as other qualitative methods.
- Participant numbers were low, which may reflect difficulties with engagement with online surveys.
- Participant representation from predominantly London and South East may not reflect practices or perspectives from other regions

## Introduction

 In England in 2025, there were 382 000 people experiencing homelessness (PEH) on any given night, with more than 4600 people sleeping rough.^1^ Homelessness is often associated with multiple physical, psychological and social barriers to seeking healthcare, and there is a strong correlation between homelessness and adverse health outcomes.^2^,^4^ Studies from the UK, USA and Australia report PEH have a 30-year lower life expectancy than the general population,^5^ alongside a significant reduction in morbidity-free life expectancy.^6^ Longitudinal studies also report 10 times higher mortality rates in this population.^7 8^ These differences could be partly attributed to the fact that primary care provision models have historically not been adapted to meet the specific needs of PEH, despite evidence on what works well for this population group.^9 10^ Further complicating matters is the fact that this populations’ complex social circumstances make follow-up or attendance difficult, so PEH can appear ‘missing’ to the health system.^11^ The rising levels of homelessness in England and the significant health inequalities faced by this population have prompted the National Health Service in England to identify homelessness as a priority action area within their broader ‘Inclusion Health’ agenda, aimed at improving access to primary care, eliminating challenges to engaging with services and improving funding for targeted programmes.^12^ These issues align with global health priorities, for example, the Street Medicine Institute focusing on facilitation of programmes to enhance the direct provision of healthcare to PEH across multiple countries in response to their unmet health needs.^13^

Point of care tests (POCTs) offer part of a strategy to improve healthcare provision.^14^ These tests are performed near the patient, typically using patient samples such as blood, urine or sputum, to provide rapid results allowing diagnosis or monitoring in real time of specific diseases and pathogens. There are many advantages to their use, including faster results leading to quicker clinical decision-making, often within the scope of one consultation, better accessibility to diagnostics and improved in-consultation collaborative management. These attributes make POCTs a promising solution to reducing barriers to healthcare and enhancing patient outcomes for PEH. Previous studies have described benefits from POCTs in this population for diabetes and blood-borne viruses (BBVs),^15^,^17^ such as enabling rapid diagnosis, prompt medication initiation, treatment adjustments and streamlining of referrals to facilitate more timely links with secondary care pathways.^18 19^ It is likely that POCTs are currently underutilised in the delivery of primary care for PEH, and with the development of new technologies the scope of POCTs is becoming increasingly broad. We, therefore, sought to explore the range of potential use cases with clinicians who care for this population.

The aim of this survey was to understand the unmet needs of primary care clinicians serving PEH, and which POCTs, either existing or not yet developed, would best aid the provision of healthcare to these individuals.

## Methods

### Development and pretesting

An anonymous web-based open survey of 21 questions was developed, with input from five researchers who had experience in health technologies research, or in clinical work with PEH. The questions asked participants about their experiences and opinions on the challenges of getting diagnostic tests performed, previous and current use of POCTs, and which POCTs could improve primary healthcare provision for this population (see online supplemental material 1). The target population was any healthcare professional providing community-based care for PEH in England. Written information regarding the purpose, expected duration, voluntary participation and data storage was provided to participants before they commenced the survey. The survey questions underwent a process of refinement by two clinicians with experience in providing primary healthcare for PEH (MW and NS) to ensure they were unambiguous and clear. The final version’s content and functionality were tested by members of the research team before it was disseminated. The study met the definition of stakeholder engagement, as it was conducted to define or judge current care provision.^20^ The Checklist for Reporting Results of Internet E-Surveys (CHERRIES) checklist was used to guide the reporting of this study.^21^

### Recruitment and survey administration

The link to the survey was circulated in March and April 2024 editions of ‘Homeless Health News’, an e-mail newsletter with a circulation of over 1600 practitioners who have an interest in homeless health in the UK, and ‘Pathway’ e-mail newsletter in April 2024 with a circulation of 2000 professionals who are interested in the field of inclusion health. Details of specific homeless healthcare providers were found via each Integrated Care Board (ICB) website, and email invitations were sent directly with links to the webpage. Participation was voluntary. A £20 e-voucher could be accessed by respondents on completion of the survey by emailing the main researcher from an NHS email, therefore keeping the responses and participant emails uncoupled and upholding participant anonymity. The survey ran from 1 March until 30 April 2024. Written information regarding the purpose, expected duration, voluntary participation was provided to participants before they commenced the survey. Randomisation of survey questions was not used. Adaptive questioning via display logic was used two times to facilitate additional inputting of free-text depending on the response to previous questions, with the aim of improving response quality.^22^ Two to three questions were displayed per screen, with the survey extending to between seven and nine screens, depending on responses to adaptive questioning. All set questions provided the option of a non-response, with no review step included for responses. No cookies or Internet Protocol (IP) checks were used to prevent multiple submissions.

### Analysis

We planned to include all survey responses regardless of level of completion. Descriptive analyses were performed using standard statistical parameters. A reflexive thematic analysis was performed for the free text responses.^23^ Inductive coding was undertaken by FR (a GP registrar and academic clinical fellow with training in qualitative research methods). Coding of the data was performed using NVivo V.14. The generated codes were discussed with members of the research team. New thematic clusters were generated, discussed and regenerated iteratively*,* to provide interpretive constructs capturing insights from the data. This was performed through a process of reflection and data immersion. These were checked back against the data to ensure they were reflective of participants' own language and experience, before production of the finalised four themes.

### Patient and public involvement

We did not involve patients or the public in this study, however it was designed and conducted with engagement of clinicians with experience of caring for PEH.

## Results

### Sample

**Figure 1:**
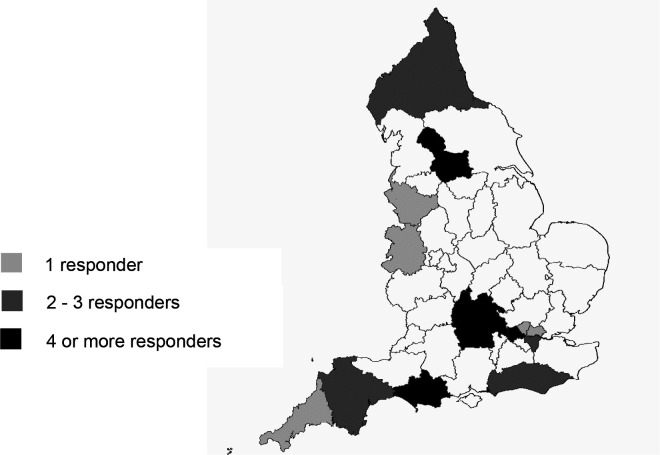
Map of showing number of survey responders by Integrated Care Boards.

A total of 32 healthcare workers participated, including 14 GPs, 9 nurses and smaller numbers of addiction workers, mental health workers and care navigators. All submitted questionnaires were fully completed, with an average completion time of 21 min and 3 s. All the responders provided answers that would be in-keeping with their claimed role as a healthcare professional. There was a reasonable geographic spread across England from responders, with 9 working for London ICBs, 14 for ICBs in the South of England, 2 in the Midlands and 7 from ICBs in the north of the country (figure 1).

Responders worked in a mixture of settings, including five in a mainstream GP practice who provided care for PEH alongside other patients, 16 from specialist GP surgeries dedicated to PEH, 7 working in community healthcare trusts and 4 in the charity sector. Specialist GP services had between 350 and 1400 service users, with charity and community services reporting smaller numbers (30 to 150 patients). Responders reported a wide range of clinical areas of special interest in their work with PEH, including drug and alcohol dependence, mental health, diabetes and women’s health (table 1). These were areas where they had undertaken additional clinical training, or were clinical leads for their service.

**Table 1:** Clinical areas of special interest reported by participants

Clinical area of special interest	Number of responses
Drug misuse	19
Alcohol dependence	18
Severe mental illness	14
Diabetes	6
Wound care	6
Cardiovascular disease	4
Respiratory disease	3
Antibiotic stewardship	3
Dermatology	2
Anticoagulant monitoring	1
Other	8

### Challenges of performing diagnostic tests

Multiple challenges were reported around performing diagnostic tests for PEH, some of which were unique to those undertaken at the primary care location itself, while others were related to investigations where referral to an external provider was required (including external diagnostic centres, endoscopy units and hospital settings). For diagnostic tests performed in primary care, 43% ‘usually’ or ‘always’ happened in the same consultation as they were requested by a clinician, with 37% ‘usually’ or ‘always’ happening in a different appointment. 29 out of 32 responders expressed that delays introduced by not having rapid access to diagnostic test results within a consultation led to significant difficulties when formulating management plans.

There were specific challenges in performing diagnostic tests, which required referral to external centres. Patient-related factors were mentioned, including issues with getting to the appointment due to financial and geographical barriers, ‘digital exclusion’ (meaning telephone appointment notifications cannot always be communicated), and fluctuation in patients’ motivation and external pressures, which influence how they prioritise their health. Logistical and organisational factors related to secondary care settings were particularly emphasised as challenging. Many participants reported a lack of flexibility in referral policies, such as secondary care providers returning referrals to GPs after patients had missed one appointment and insisting on the use of online portals to confirm appointments, which is incompatible with the circumstances of individuals experiencing digital exclusion. Negative staff attitudes in secondary care towards this group, patients feeling overwhelmed by the surroundings as well as a lack of time to build trust in the service, meant that patients were more likely to either not attend or walk out of the appointment.

For diagnostic tests performed ‘in house’ (but not using POCTs), the challenges included difficulties obtaining venous blood samples due to previous intravenous drug use, and individuals’ health beliefs and low health literacy leading them to decline tests. Responders reported that some patients were reluctant to stay longer at an appointment to have tests performed and often did not return to future appointments if these were scheduled. Staff also expressed concerns about communicating results from tests that required external processing, as the results were typically available outside of the appointment time. This was especially challenging when results indicated a need for treatment, as patients’ chaotic and transient circumstances often made follow-up communication difficult.

### Current use and experience of POCTs in homeless healthcare

**Figure 2:**
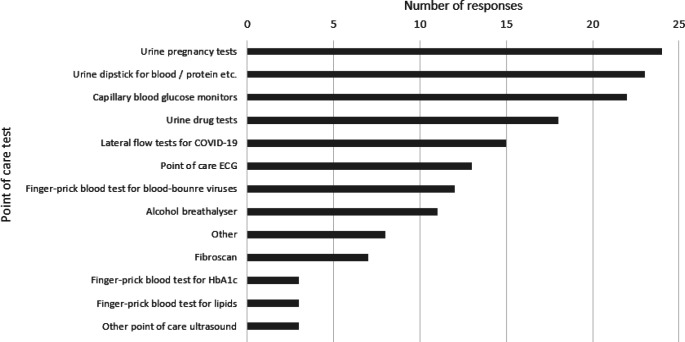
Number of responders regularly using different point of care tests in clinical practice. HbA1c, haemoglobin A1c.

Six participants (19%) reported that their service did not use any POCTs when providing healthcare to PEH. For the services where POCTs were available, the most common devices in regular use were urine dipstick, urine pregnancy tests, capillary blood glucose monitors and urine testing for recreational drugs (see figure 2).

POCTs were considered to add value to healthcare delivery by most respondents. 17 responders commented on the benefits of performing rapid tests in facilitating a discussion of diagnosis and management within the same consultation. The two situations where this was reported to be most beneficial were BBV testing and cardiometabolic risk assessments. Clinicians were able to deliver health education at a time when patients were more receptive, increasing the likelihood of sustained behaviour changes and better engagement with any medications prescribed. There were also practical benefits highlighted in terms of enabling the tests to be brought to the ‘hardest to reach’ populations, allowing some of the unmet health needs of this population to be addressed.

There were challenges reported around using POCTs in this setting. One common issue was that the technology did not have suitable functionalities, such as no wireless capability to allow for use in outreach, or not connecting with the electronic patient record leading to a time burden of manually inputting results, and with potential transcription errors. Three responders highlighted difficulties in obtaining ongoing funding as well as navigating the lengthy procurement processes to obtain new, improved equipment, with one responder linking these difficulties with a sense that improving access to diagnostics for those experiencing homelessness was not a priority in their region. Other issues raised were lack of physical space for diagnostic hardware and staff shortages leading to insufficient nursing time to carry out tests or undertake training to maintain competency in use of specialised equipment.

### POCTs of potential value

Clinical use cases suggested by responders for POCTs included C reactive protein (CRP) testing specifically for the management of respiratory tract infections where coexistent inhaled drug can complicate the clinical presentation, and POCTs for detecting wound infection in rough sleepers, a population at higher risk of wound infections.^24^ Point of care ultrasound for deep vein thrombosis (DVT) investigation was also reported to be useful. While usual care would be to initiate anticoagulation pending outpatient ultrasound confirmation or rule out, in this patient cohort, there was felt to be a risk of either lack of anticoagulant adherence or long-term anticoagulant use without diagnostic confirmation, a heightened risk given the high rates of alcohol excess.

Four participants had previously used specific POCTs in their clinical practice, but had stopped using them for a variety of reasons. POCTs for d-dimer and urine drug screens were found to not have added significant value to the consultation or changed clinician decision-making, and faecal occult blood POCTs were found to have collection methods which were not acceptable to patients leading to non-engagement.

Certain POCTs were reported to have the potential to improve chronic disease management, acute care and addiction work. 21 out of 32 participants reported that tests for cardiometabolic risk evaluation, such as lipids and haemoglobin A1c (HbA1c), would be the most useful in chronic disease as well as tests which are needed to monitor medication or assess for disease progression, such as renal and liver function tests. BBV testing, including viral genotyping, and fibroscan were the second most frequent response. For acute care, 12 responders stated CRP would be the most beneficial, closely followed by renal function (seven responders) and troponin testing (five responders). Three expressed that point of care DVT diagnostics by ultrasound would be helpful, with similar numbers selecting point of care infection swabs (respiratory, wound and STI) to facilitate reducing antibiotic prescribing. In addiction work, opinion was split, with some expressing that tests for newer synthetic street drugs, such as nitazenes and ‘Spice’, would be helpful, while others expressed that patients were often honest about what they were using, and testing could undermine the development of a trusting therapeutic relationship.

**Figure 3:**
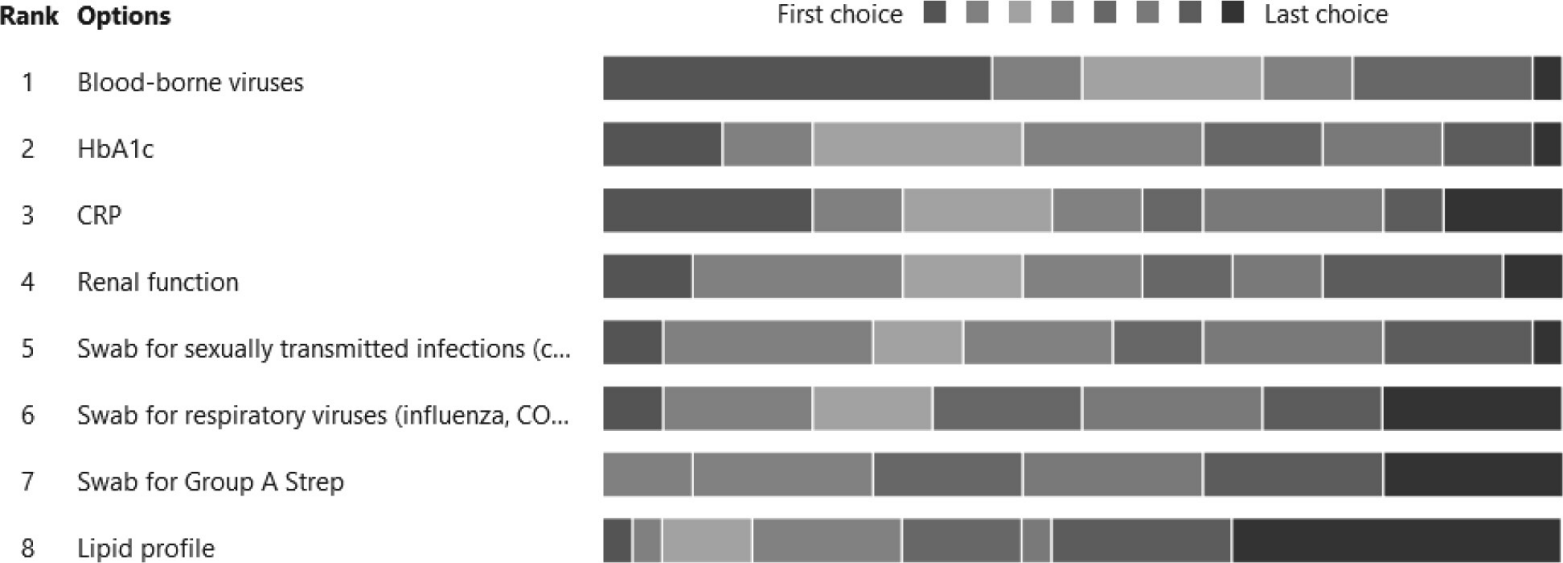
Cumulative ranking for point of care tests that would most improve primary care for people experiencing homelessness. HbA1c, haemoglobin A1c; CRP, C reactive protein; Group A Strep, Group A Streptococcus.

Figure 3 shows the overall cumulative ranking for eight POCTs, which responders reported would most improve primary care for PEH. 41% of responders ranked BBV testing highest, with lipid profile ranked as least useful.

### Unmet healthcare needs for technology

Finally, responders were asked which other diagnostic or disease management challenges could be helpfully addressed by a POCT from their experience of working with PEH. Responders were encouraged to suggest areas even if they were unsure if there was a POCT available currently. The three most commonly given responses were malnutrition and vitamin testing (such as iron, B12 and vitamin C and D), chest X-rays and TB testing, and simple point of care lung function testing to detect COPD, often underdiagnosed in this cohort. Other suggestions were POCTs for cancer screening, neurodiversity and traumatic brain injury diagnosis, and tests to differentiate epileptic seizures from other causes of seizure-type activity.

## Discussion

This survey has identified a range of situations where POCTs could assist in primary care provision for PEH. Multiple challenges in performing clinical investigations for PEH were described, which could broadly be classified into resource problems, such as equipment availability, staff availability and limited funding and patient factors, such as communication, digital exclusion and geographic or financial barriers. 91% of healthcare workers who responded to this survey commented that delays from not having access to rapid diagnostic tests in primary care led to significant difficulties in consultations. This can lead to missed opportunities to provide timely clinical management. POCTs were considered a solution to this issue, but currently there is significant variation in test availability across services and regions. Testing for BBVs and cardiometabolic risk was considered to provide most overall benefit to patients. Tests to evaluate underlying cardiometabolic risk, renal and liver profiles were rated as most useful in the management of chronic disease, whereas CRP was rated most beneficial in acute care. Functionality of specific devices is important to consider before implementation, such as their suitability for use in outreach work and integration into current workflows.

### Strengths and limitations

While the survey was designed with input from multiple researchers with health technologies and inclusion healthcare clinical and research experience, it would have been better to coproduce the questionnaire with input from those with lived experience of homelessness, to gain alignment with patient priorities and ensuring approachability and acceptability of POCTs have been considered. The survey was open to all healthcare workers who have experience of providing primary healthcare to PEH, providing an overview of potential POCT utilisation. The responders provided a sample that reflects different job roles across the inclusion health workforce in primary care. However, the appreciation of the role of specific tests will be different between staff groups, as, for example, some GPs may have less appreciation of how POCTs fit with addiction management, and conversely addiction workers may not consider how they could help in the management of possible DVTs. Therefore, simply considering the frequency with which certain responses were given will not provide the full picture of potential POCT uses. The survey also collected responses of positive and negative experiences of POCTs, providing an opportunity for balance relating to their potential use. Another strength was the survey distribution strategy, which used multiple methods and networks for dissemination. However, despite the use of evidence-based approaches to improve response rates, such as offering financial remuneration and ensuring good formatting via pilot testing,^25^ the number of responses was small with a disproportionate number from London and the South of England. This could potentially risk inadequate representation within the data, however, as not many clinicians deliver this specific type of healthcare, a large response rate was not anticipated. A further limitation was that it was not possible to determine if multiple people from certain organisations or services completed the survey, therefore over-representing certain opinions and experiences, potentially reducing transferability. However, the benefits of maintaining responder anonymity, such as promoting authentic voices from participants, need to be weighed against this.

### Comparison with literature

The concept of access to healthcare requires consideration at multiple levels, including individual, community, provider and healthcare system factors.^26^ Levesque’s model of access to healthcare, one of the most frequently utilised models, explains that access depends on both the healthcare system (such as approachability and availability) and the patient’s abilities (like their ability to perceive, seek, reach and engage with care).^27^ The utilisation of POCTs for PEH addresses three of the five dimensions of the model: availability by improving timely access to diagnostics, affordability by reducing the need to attend for multiple appointments and appropriateness by ensuring there is alignment between services provided and the specific needs of the client population. All of these factors align with perspectives from survey responders on the benefits of POCT, emphasising their potential role for this under-served population.

POCTs are currently mostly being used for BBVs for PEH. Evaluation of these tests has shown high levels of acceptability and feasibility for patients and healthcare providers,^28^ with notable reduction in time from test to treatment initiation and increased treatment uptake.^29^ This matches the survey responses, where clinicians expressed that there would be a role for POCTs, especially in facilitating diagnosis within one consultation. However, there is very little in the literature evaluating other POCTs for PEH, including CRP. Half of the survey participants reported that CRP would be the most beneficial POCT in acute care provision. The main justification was to improve antimicrobial stewardship, which would be advantageous as homeless healthcare providers have been shown to prescribe more antibiotics than mainstream general practice settings in England.^8^ CRP testing in the community has been effectively incorporated into clinical guidelines in many countries (including Norway, Denmark and Spain), most commonly for clinical decision-making related to respiratory tract infections.^30^ This has not been replicated in the UK, where barriers include issues of funding and reimbursement, concerns around over-reliance and uncertainty related to test integration into practice workflow.^31^ In order for CRP POCTs to provide benefit for PEH, new guidelines are likely to be required that consider their unique healthcare circumstances, such as the risk of non-return in cases of clinical deterioration.

Ascertaining cardiometabolic risk is increasingly important due to changing demographics of homeless populations. The Office of National Statistics census in 2021 revealed that 10% of women and 19% of men experiencing homelessness were aged 50 and over, with a steady increase in average age since 2001.^32^ With this increasing age comes an increasing number of chronic health conditions and medical complexity. Data from the Glasgow Homeless Health Service found that their average patient had levels of multimorbidity comparable to those aged ≥85 years in the general population,^33^ despite the average age being 43 years. While POCTs for cardiometabolic risk factors have the potential to facilitate diagnosis, improve disease control and initiate educational interactions, their availability would ideally be coupled with development of healthcare staff expertise in tailored clinical management, taking their housing circumstances into account. There are also high levels of medication non-adherence in this population, with one study finding only 25% of patients were taking appropriate secondary prevention medications post myocardial infarction.^33 34^ This highlights how it is not just the provision of the tests, but considering how these fit with patient care as a whole that is paramount to successful implementation and clinical impact.

### Implications for research and practice

The survey responses outline factors which contribute to stopping POCT use, which provides insights into some of their pitfalls in clinical practice. A key factor for successful POCT implementation is clinician understanding of how the test result would influence the consultation outcome. Without this, a POCT is unlikely to be widely adopted. Further research would be needed to ascertain which POCTs should be performed proactively (more like screening tools), and which reactively based on patient symptoms, in order to provide the most clinical benefit. Incorporating performing a test into consultations will require training and consideration of the time allotted to each patient contact, but could be managed by certain members of staff being responsible for diagnostics delivery.

Specific areas were suggested where further research into POCTs for PEH would be beneficial. These broadly fell into two categories; those where POCTs are already available and the focus would be their implementation in this setting, and others which were ‘aspirational’, where the tests themselves require development. POC ultrasound has been shown to aid the diagnosis of many respiratory pathologies in primary care, such as pneumonia and pleural effusion,^35^ and is also used in the diagnosis of DVT. However, implementation in this context would be a challenge, from both a financial and logistical perspective due to equipment costs, staff training and ongoing accreditation of clinicians to use it safely and effectively. Research would be required to understand how these factors balance against potential patient benefits, including the difficulties with secondary care access for PEH.^36^ In relation to wound care, there are an increasing number of POCT devices for infection detection and monitoring of healing,^37^ where studies could explore the cost-effectiveness of implementation. This is in contrast to POCTs for nutritional deficiencies (especially vitamin C, D and B12 levels) and neurodiversity, which, while healthcare workers reported a desire for them, are not currently available, highlighting these as priorities for development.

### Conclusion

Although there was evidence of POCT use by healthcare providers and primary healthcare to PEH, there was variation across services as to which tests are available. The vast majority of responders reported that enhancing the provision of POCTs would be beneficial, both in acute and chronic care scenarios, due to the benefits of getting rapid results and reducing the need for repeat appointments or onward referral for diagnostics.

## Supplementary material

10.1136/bmjopen-2025-113834 online supplemental file 1

## Data Availability

Data are available upon reasonable request.
