# Supplementary material for: Perspectives of clinicians caring for people experiencing homelessness on point of care tests: an online survey
Source: BMJ Open. 2026 Jul 16;16(7):e113834. doi: 10.1136/bmjopen-2025-113834 (PMC13384143; doi:10.1136/bmjopen-2025-113834)
Supplement: online supplemental file 1 [file bmjopen-16-7-s001.docx]

**Appendix 1: Copy of Survey used for data collection**

1. **What is your current clinical role? *(select one only)***
   1. GP
   2. Nurse
   3. HCA
   4. Addiction worker
   5. Mental health worker
   6. Social prescriber
   7. Other – please specify (free text)
2. **Do you have a clinical area of special interest (e.g. have undertaken extra training, or are a clinical lead) in your work with patients experiencing homelessness? *(choose all that apply)***
   1. Severe mental illness
   2. Drug misuse
   3. Alcohol dependence
   4. Diabetes
   5. Respiratory disease – Asthma / COPD
   6. Cardiovascular disease
   7. Antibiotic stewardship
   8. Skin conditions
   9. Wound care
   10. Anticoagulant monitoring
   11. Other (specify) *(free text)*
3. **Which Integrated Care System do you work under (ICS)?** *(select one)*
4. **Do you work at: *(select one)***
5. A specialist GP surgery only for people experiencing homelessness
6. A regular GP surgery who also registers homeless people
7. **Can you provide an estimate of the number of patients experiencing homelessness registered at your surgery? *(free text)***
8. **What are the challenges of undertaking diagnostic tests which are performed “in house” for patients experiencing homelessness? *(free text)***
9. **What are the challenges of undertaking diagnostic tests whc=ich require external referrl for patients experiencing homelessness? (*free text)***
10. **For diagnostic tests which do not need an external referral, how often are these performed in:**
    1. the same consultation

1 (never), 2 (rarely), 3 (half the time), 4 (often), 5 (always)

- 1. the same location, but a separate appointment

1 (never), 2 (rarely), 3 (half the time), 4 (often), 5 (always)

1. **For patients experiencing homelessness, are there specific difficulties in formulating a management plan as a result of waiting for diagnostic tests to be performed and results to be received?** *(Yes/No)*
2. **If so, what are these challenges?** *(free text)*
3. **Are you currently using any of these POCTs in your homeless healthcare delivery?**
   - 1. Urine dipsticks for blood / protein / leucocytes / nitrites / glucose
     2. Urine drug tests
     3. Urine pregnancy tests
     4. Capillary blood glucose monitors
     5. Lateral flow tests for COVID-19
     6. Lateral flow tests for Group A Strep
     7. Alcohol breathalyser
     8. Finger-prick blood tests for blood-borne viruses (Hep B / Hep C / HIV)
     9. Finger-prick blood test for CRP
     10. Other finger-prick blood tests e.g. HbA1c or lipid profile (please specify)
     11. Point of care ECG
     12. Fibroscan
     13. Other point of care ultrasound tests (please specify)
     14. Other tests (please specify)
     15. No, we use no POC devices
4. **Which features of these tests do you find most valuable in clinical practice? *(free text)***
5. **Have you previously tried using any other POCTs in your homeless healthcare delivery, but have chosen to stop using them? (Yes/No)**
   1. If yes, which ones? *(choose all that apply)*
      1. Urine dipsticks for blood / protein / leucocytes / nitrites
      2. Urine drug tests
      3. Urine pregnancy tests
      4. Capillary blood glucose monitors
      5. Lateral flow tests for COVID-19
      6. Lateral flow tests for Group A Strep
      7. Alcohol breathalyser
      8. Finger-prick blood tests for blood-borne viruses (Hep B / Hep C / HIV)
      9. Finger-prick blood test for CRP
      10. Other finger-prick blood tests e.g. HbA1c or lipid profile (please specify)
      11. Point of care ECG
      12. Fibroscan
      13. Other point of care ultrasound tests (please specify)
      14. Other tests (please specify)
6. **If you answered yes to question 12, can you explain why you chose to stop using these POCTs? (free text)**
7. **Availability of which POC tests do you feel would most improve care for people experiencing homelessness? (rank in order of importance, 1 being most important, 8 being least important)**
   1. CRP
   2. Renal function
   3. HbA1c
   4. Lipid profile
   5. Blood-borne viruses
   6. Swab for Group A Strep
   7. Swab for respiratory viruses (influenza, COVID-19, RSV)
   8. Swab for sexually transmitted infections (chlamydia, gonorrhoea)
   9. Other test not listed above (specify)
8. **Which POC tests would be most helpful when carrying out urgent primary care for those experiencing homelessness? (free text)**
9. **Which POC tests would be most helpful when managing chronic diseases for those experiencing homelessness? (free text)**
10. **Which POC tests would be most helpful when carrying out substance misuse management for those experiencing homelessness? (free text)**
11. **What do you think could be barriers to using a POCT for people experiencing homelessness (may include patient, system and clinician related barriers)?** (*free text)*
12. **In your usual clinical practice working with people experiencing homelessness, which other diagnostic or management challenges could be addressed by a POCT? [Please suggest any important areas, even if a POCT does not currently exist for this]** (*free text)*

**Appendix 2: Survey distribution wording**

*Newsletter advertisement*

**Point of Care Testing in Homeless Healthcare**

We would like to understand how new technologies and point of care testing could be used to improve the provision of primary care for people experiencing homelessness. We are carrying out a survey of healthcare professionals who work with this population, to gauge opinion as to what tests and technologies would be most valuable, as well as improve our understanding of the potential barriers and challenges involved. This will hopefully lead to piloting new devices which could help provide better and more efficient care for patients.

The survey can be found via this link: XXXXX

A £20 e-voucher will be provided on completion of the survey for reimbursement of your time.

*Email invitation*

Hello,

We are a team of researchers and clinicians at Oxford University, who would like to understand how new technologies and point of care testing could be used to improve the provision of primary healthcare for people experiencing homelessness. We are carrying out a survey of healthcare professionals who work with this population, to gauge opinion as to what tests and technologies would be most valuable, as well as improve our understanding of the potential barriers and challenges involved. This will hopefully lead to piloting new devices which could help provide better and more efficient care for patients.

The survey can be found via this link: XXXXX

A £20 e-voucher will be provided on completion of the survey for reimbursement of your time (instructions on how to do this at the beginning of the survey).

We would be really grateful if you could circulate this to your healthcare staff who work with you for the homeless population so we could understand their views.

Any questions, please let me know

**Appendix 3: CHERRIES checklist for reporting**

| *Item Category* | *Checklist Item Explanation* | *Location in paper (pg)* |
| --- | --- | --- |
| **Design** | | |
| Describe survey design | Describe target population, sample frame. Is the sample a convenience sample? (In “open” surveys this is most likely.) | 5 |
| **IRB (Institutional Review Board) approval and informed consent process** | | |
| IRB approval | Mention whether the study has been approved by an IRB | 5 |
| Informed consent | Describe the informed consent process. Where were the participants told the length of time of the survey, which data were stored and where and for how long, who the investigator was, and the purpose of the study? | 5, 6 |
| Data protection | If any personal information was collected or stored, describe what mechanisms were used to protect unauthorized access | 5 |
| **Development and pre-testing** | | |
| Development and testing | State how the survey was developed, including whether the usability and technical functionality of the electronic questionnaire had been tested before fielding the questionnaire | 5 |
| **Recruitment process and description of the sample having access to the questionnaire** | | |
| Open survey versus closed survey | An “open survey” is a survey open for each visitor of a site, while a closed survey is only open to a sample which the investigator knows (password-protected survey). | 5 |
| Contact mode | Indicate whether or not the initial contact with the potential participants was made on the Internet. | 5 |
| Advertising the survey | How/where was the survey announced or advertised? Some examples are offline media (newspapers), or online (mailing lists – If yes, which ones?) or banner ads (Where were these banner ads posted and what did they look like?). It is important to know the wording of the announcement as it will heavily influence who chooses to participate. Ideally the survey announcement should be published as an appendix. | 5 |
| **Survey administration** | | |
| Web/E-mail | State the type of e-survey (e.g. one posted on a Web site, or one sent out through e-mail). If it is an e-mail survey, were the responses entered manually into a database, or was there an automatic method for capturing responses? | 5 |
| Context | Describe the Web site (for mailing list/newsgroup) in which the survey was posted. What is the Web site about, who is visiting it, what are visitors normally looking for? Discuss to what degree the content of the Web site could pre-select the sample or influence the results. For example, a survey about vaccination on an anti-immunization Web site will have different results from a Web survey conducted on a government Web site | 5 |
| Mandatory/voluntary | Was it a mandatory survey to be filled in by every visitor who wanted to enter the Web site, or was it a voluntary survey? | 6 |
| Incentives | Were any incentives offered (e.g. monetary, prizes, or non-monetary incentives such as an offer to provide the survey results)? | 5 |
| Time/Date | In what timeframe were the data collected? | 6 |
| Randomization of items or questionnaires | To prevent biases items can be randomized or alternated. | 6 |
| Adaptive questioning | Use adaptive questioning (certain items, or only conditionally displayed based on responses to other items) to reduce number and complexity of the questions. | 6 |
| Number of Items | What was the number of questionnaire items per page? The number of items is an important factor for the completion rate. | 6 |
| Number of screens (pages) | Over how many pages was the questionnaire distributed? The number of items is an important factor for the completion rate. | 6 |
| Completeness check | It is technically possible to do consistency or completeness checks before the questionnaire is submitted. Was this done, and if “yes”, how (usually JAVAScript)? An alternative is to check for completeness after the questionnaire has been submitted (and highlight mandatory items). If this has been done, it should be reported. All items should provide a non-response option such as “not applicable” or “rather not say”, and selection of one response option should be enforced. | 6 |
| Review step | State whether respondents were able to review and change their answers (e.g. through a Back button or a Review step which displays a summary of the responses and asks the respondents if they are correct). | 6 |
| **Response rates** | | |
| Unique site visitor | If you provide view rates or participation rates, you need to define how you determined a unique visitor. There are different techniques available, based on IP addresses or cookies or both | 6 |
| View rate (Ratio of unique survey visitors/unique site visitors) | Requires counting unique visitors to the first page of the survey, divided by the number of unique site visitors (not page views!). It is not unusual to have view rates of less than 0.1 % if the survey is voluntary. | N/A |
| Participation rate (Ratio of unique visitors who agreed to participate/unique first survey page visitors) | Count the unique number of people who filled in the first survey page (or agreed to participate, for example by checking a checkbox), divided by visitors who visit the first page of the survey (or the informed consents page, if present). This can also be called “recruitment” rate | N/A |
| Completion rate (Ratio of users who finished the survey/users who agreed to participate) | The number of people submitting the last questionnaire page, divided by the number of people who agreed to participate (or submitted the first survey page). This is only relevant if there is a separate “informed consent” page or if the survey goes over several pages. This is a measure for attrition. Note that “completion” can involve leaving questionnaire items blank. This is not a measure for how completely questionnaires were filled in. (If you need a measure for this, use the word “completeness rate”.) | N/A |
| **Preventing multiple entries from the same individual** | | |
| Cookies used | Indicate whether cookies were used to assign a unique user identifier to each client computer. If so, mention the page on which the cookie was set and read, and how long the cookie was valid. Were duplicate Not used entries avoided by preventing users access to the survey twice; or were duplicate database entries having the same user ID eliminated before analysis? In the latter case, which entries were kept for analysis (e.g. the first entry or the most recent)? | 6 |
| IP check | Indicate whether the IP address of the client computer was used to identify potential duplicate entries from the same user. If so, mention the period of time for which no two entries from the same IP address were allowed (e.g. 24 hours). Were duplicate entries avoided by preventing users with the same IP address access to the survey twice; or were duplicate database entries having the same IP address within a given period of time eliminated before analysis? If the latter, which entries were kept for analysis (e.g. the first entry or the most recent)? | 6 |
| Log file analysis | Indicate whether other techniques to analyse the log file for identification of multiple entries were used. If so, please describe. | N/A |
| Registration | In “closed” (non-open) surveys, users need to login first and it is easier to prevent duplicate entries from the same user. Describe how this was done. For example, was the survey never displayed a second time once the user had filled it in, or was the username stored together with the survey results and later eliminated? If the latter, which entries were kept for analysis (e.g. the first entry or the most recent)? | N/A |
| **Analysis** | | |
| Handling of incomplete questionnaires | Were only completed questionnaires analysed? Were questionnaires which terminated early (where, for example, users did not go through all questionnaire pages) also analysed? | 6 |
| Questionnaires submitted with an atypical timestamp | Some investigators may measure the time people needed to fill in a questionnaire and exclude questionnaires that were submitted too soon. Specify the timeframe that was used as a cut-off point, and describe how this point was determined. | N/A |
| Statistical correction | Indicate whether any methods such as weighting of items or propensity scores have been used to adjust for the non-representative sample; if so, please describe the methods. | N/A |
