# Supplementary material for: Perspectives of clinicians caring for people experiencing homelessness on point of care tests: an online survey
Source: BMJ Open. 2026 Jul 16;16(7):e113834. doi: 10.1136/bmjopen-2025-113834 (PMC13384143; doi:10.1136/bmjopen-2025-113834)
Supplement: Supplementary file 2 [file bmjopen-2025-113834.reviewer_comments.pdf]

# PEER REVIEW HISTORY

BMJ Open publishes all reviews undertaken for accepted manuscripts. Reviewers are asked to complete a checklist review form and are provided with free text boxes to elaborate on their assessment. These free text comments are reproduced below.

## ARTICLE DETAILS

**Title (Provisional)**

Perspectives of clinicians caring for people experiencing homelessness on point of care tests: an online survey

**Authors**

Rose, Frances; Ukwatte, Umasha; Singh, Neil; Turner, Philip J; Willcox, Merlin; Lennox, Belinda; Hayward, Gail N

---

## VERSION 1 - REVIEW

---

| | |
|---|---|
| **Reviewer** | **1** |
| **Name** | **Martin-Misener, Ruth** |
| **Affiliation** | **Dalhousie University** |
| **Date** | **01-Jan-2026** |
| **COI** | **None** |

---

1. Well written article about an important issue.

2. More detail is needed about the development of the questionnaire for the survey and the steps taken to ensure question clarity, content validity and reliability.

3. Provide a brief description of adaptive questioning with an example and a reference

4. Definition of stakeholder engagement on page 6 needs a reference.

5. Was it verified with an Research Ethics Board that ethics approval was not required?

6. Please provide more detail about how reflexive thematic analysis and inductive coding were applied and who was involved and what assessments or processes were used to ensure quality in the coding process?

7. Need a reference on page 10 for the statement that rough sleepers have higher risk of wound infection

8. Use of the expression “were felt” when reporting qualitative data is problematic since we do not know how respondents felt only what they expressed/reported, For example on page 7, lines 46-49

9. Line 45 on page 7 begins with a number written numerically. Since it is the beginning of a sentence it should be written as a word.

10. Several figures use acronyms that should be defined in the accompanying title

11. The article reports that the CHERRIES checklist was used, however, the checklist is not included with the submission

---

| | |
|---|---|
| **Reviewer** | **2** |
| **Name** | **Williamson, Andrea** |
| **Affiliation** | **University of Glasgow** |
| **Date** | **28-Jan-2026** |
| **COI** | **None** |

---

It is good to see some research scrutiny of an important aspect of Inclusion Health practice.

I understand why online survey methods were chosen to help engage busy professionals, however on reading your results I was left wondering why participants described some of what had changed in their practice. I do agree that the numbers are too low to comment on and I am left thinking how that can be represented in your results? Is there a theoretical approach that can help with how you report your results like Levesque's access framework for example? I suggest having a think about that and considering a re-write based on this.

Also more information is required regarding the RTA you used and this gives you ample scope to include the theoretical perspective suggested.

I have some other more minor comments to make which will improve the next version of the paper also:

Abstract

poorer outcomes may be driven by barriers to care but we do not know that- delete 'consequently'

Avoid words like 'vast' to describe majority when reporting scientific results

Strengths and limitations

survey addresses 'care' for an important population

I don't think your justification of low numbers being low numbers in the field can be included as you describe quite high numbers of professionals being contacted.

Main text

Introduction

p4 47 Please use current stats on prevalence of homelessness rather than a 5 year old paper. Shelter reports on this and its publically available, use England stats as this study covers England.

p5 3 not clear what 'social travails' means - maybe say complex social circumstances?

p5 18 I would say England here rather than UK as health and housing are devolved. Also important to cite the NICE Guideline in terms of evidence base and policy response (NG 214).

P5 19 I think POCTs offer 'part' of the solution

p7 52 'mainly related to resource and patient related factors' is too broad a statement to be helpful. Please revisit this- using a theoretical framing might help with this.

p8 52 this section needs edited to distinguish between those who had no POCTs access and what was used.

p11 8, explain why this study should have been co-produced with experts by experience?

p11 12 suggest avoid using representative when not talking stats representative in this sort of research context,suggest 'sample that reflects' instead.

p11 32 again using stats related words - revisit this.

p12 suggest saying women and men rather than females and males in this research context.

---

## VERSION 1 - AUTHOR RESPONSE

Reviewer: 1

Comments to the Author:
1. Well written article about an important issue.

2. More detail is needed about the development of the questionnaire for the survey and the steps taken to ensure question clarity, content validity and reliability.

*Thank you, the following statement has been added to address the questionnaire development "The survey questions were refined by two clinicians with experience in providing primary healthcare for PEH (MW and NS) to ensure they were unambiguous and clear.*

*Validity and reliability are less important for this survey, as the aim was to gain a snapshot of current views, perspectives and experiences, rather than a fully comprehensive qualitative study.*

3. Provide a brief description of adaptive questioning with an example and a reference

*We have edited to now read "Adaptive questioning via display logic was used twice to facilitate additional inputting of free-text depending on the response to previous questions, with the aim of improving response quality." The following reference has also been added "Williams RT, Ingleby E. The online survey in qualitative research: can AI act as a probing tool? Front Res Metr Anal. 2025 Sep 5;10:1519008. doi: 10.3389/frma.2025.1519008. PMID: 40977760; PMCID: PMC12447317." which discusses adaptive questioning, and its merits for online surveys in qualitative research.*

4. Definition of stakeholder engagement on page 6 needs a reference.

*We added a definition "the study met the definition of stakeholder engagement, as it was conducted to define or judge current care provision, meaning that ethical approval was not required" and included reference to the NHS Health Research Authority decision tool for defining research.*

5. Was it verified with an Research Ethics Board that ethics approval was not required?

*Yes – surveys were sent to the University of Oxford Medical Sciences Interdivisional Research Ethics Committee, who approve them and stated that ethics would not be required.*

6. Please provide more detail about how reflexive thematic analysis and inductive coding were applied and who was involved and what assessments or processes were used to ensure quality in the coding process?

*We have expanded on this section, which now reads "Inductive coding was undertaken by FR (a GP registrar and academic clinical fellow with training in qualitative research methods). Coding of the data was performed using NVivo 14. The generated codes were discussed with members of the research team. New thematic clusters were generated, discussed and re-generated iteratively, to provide interpretive constructs capturing insights from the data. This was performed through a process of reflection and data immersion in the data. These were checked back against the data to ensure they were reflective of participants own language and experience, before production of the finalised four themes."*

7. Need a reference on page 10 for the statement that rough sleepers have higher risk of wound infection

*We have now included this reference: Stephens M, Chester-Bessell D, Rose S. Wound care in hard-to-reach populations: rough sleepers. Br J Nurs. 2024 Feb 22;33(4):S34-S37. doi: 10.12968/bjon.2024.33.4.S34. PMID: 38386529.*

8. Use of the expression "were felt" when reporting qualitative data is problematic since we do not know how respondents felt only what they expressed/reported, For example on page 7, lines 46-49

*Thank you, “felt” has been changed throughout to expressed, reported or considered.*

9. Line 45 on page 7 begins with a number written numerically. Since it is the beginning of a sentence it should be written as a word.

*Thank you, this has been corrected.*

10. Several figures use acronyms that should be defined in the accompanying title

*These have been expanded in the figure legends.*

11. The article reports that the CHERRIES checklist was used, however, the checklist is not included with the submission

*We have now included this as an appendix with the submission, thank you.*

Reviewer: 2
Dr. Andrea Williamson, University of Glasgow
Comments to the Author:
It is good to see some research scrutiny of an important aspect of Inclusion Health practice. I understand why online survey methods were chosen to help engage busy professionals, however on reading your results I was left wondering why participants described some of what had changed in their practice. I do agree that the numbers are too low to comment on and I am left thinking how that can be represented in your results? Is there a theoretical approach that can help with how you report your results like Levesque's access framework for example? I suggest having a think about that and considering a re-write based on this. Also more information is required regarding the RTA you used and this gives you ample scope to include the theoretical perspective suggested.

*Thank you for your comments. We have added a paragraph to the discussion section relating to Leveque’s access framework, as follows:*

*“The concept of access to healthcare requires consideration at multiple levels, including individual, community, provider and healthcare system factors.[26] Levesque’s model of access to healthcare, one of the most frequently utilised models, explains that access depends on both the healthcare system (such as approachability and availability) and the patient’s abilities (like their ability to perceive, seek, reach and engage with care).[27] The utilisation of POCTs for PEH addresses three of the five dimensions of the model: availability by improving timely access to diagnostics, affordability by reducing the need to attend for multiple appointments, and appropriateness by ensuring there is alignment between services provided and the specific needs of the client population. All of these factors align with*

*perspectives from survey responders on the benefits of POCTs, emphasising their potential role for this under-served population."*

*However, we do not feel that a re-write is needed using the framework. Multiple aspects of the framework are from the perspective of the service users of the healthcare system compared to our survey which focused on the perspectives, opinions and experiences of clinicians, meaning many aspects of the framework could not be commented on with the data collected.*

I have some other more minor comments to make which will improve the next version of the paper also:
Abstract

1. Poorer outcomes may be driven by barriers to care but we do not know that- delete 'consequently'

*Removed.*

2. Avoid words like 'vast' to describe majority when reporting scientific results

*Removed*

Strengths and limitations
3. survey addresses 'care' for an important population

*Amended*

I don't think your justification of low numbers being low numbers in the field can be included as you describe quite high numbers of professionals being contacted.

*This has been revised to "Participant numbers were low, which may reflect difficulties with engagement with online surveys"*

Main text
Introduction
p4 47 Please use current stats on prevalence of homelessness rather than a 5 year old paper. Shelter reports on this and its publically available, use England stats as this study covers England.

*Thank you – this has been amended to "In England in 2025, there were 382,000 people experiencing homelessness (PEH) on any given night, with more than 4,600 people sleeping rough" and including the Homelessness in England Report from Shelter 2025 as a reference (*FINAL_-_SHELTER_-_HOMELESSNESS_IN_ENGLAND_2025.pdf)

p5 3 not clear what 'social travails' means - maybe say complex social circumstances?

*This has been changed to complex social circumstances.*

p5 18 I would say England here rather than UK as health and housing are devolved. Also important to cite the NICE Guideline in terms of evidence base and policy response (NG 214).

*Thank you – this has been edited and the NICE Guidelines referenced*

P5 19 I think POCTs offer 'part' of the solution

*Amended*

p7 52 'mainly related to resource and patient related factors' is too broad a statement to be helpful. Please revisit this- using a theoretical framing might help with this.

*Thank you – this has been addressed by re-writing the section on challenges of performing diagnostic tests for PEH. The section has been broken down to improve clarity over which challenges relate to POCTs and which related to other in house tests, and generic wording removed.*

p8 52 this section needs edited to distinguish between those who had no POCTs access and what was used.

*"For the services where POCTs were available, the most common devices in regular use were urine dipstick..." has been added for clarity to differentiate between the sections.*

p11 8, explain why this study should have been co-produced with experts by experience?

*We have added "to gain alignment with patient priorities, and ensuring approachability and acceptability of POCTs have been considered." to expand on the above point*

p11 12 suggest avoid using representative when not talking stats representative in this sort of research context, suggest 'sample that reflects' instead.

*This has been amended – thank you.*

p11 32 again using stats related words - revisit this.

*This has been revised, and so now does not feature quantitative data terminology.*

*"However, despite the use of evidence-based approaches to improve response rates, such as offering financial remuneration and ensuring good formatting via pilot testing,[21] the number of responses was small with a disproportionate number from London and the South*

*of England. This could potentially risk inadequate representation within the data, or not reaching theme saturation and therefore being unable to gain meaningful insights and in-depth understanding, however as not many clinicians deliver this specific type of healthcare a large response rate was not anticipated. A further limitation was that it was not possible to determine if multiple people from certain organisations or services completed the survey, therefore over-representing certain opinions and experiences, potentially reducing transferability. However, the benefits of maintaining responder anonymity, such as promoting authentic voices from participants, need to be weighed against this."*

p12 suggest saying women and men rather than females and males in this research context.

*Thank you – this has been changed.*

---

## VERSION 2 - REVIEW

---

| | |
|---|---|
| **Reviewer** | **2** |
| **Name** | **Williamson, Andrea** |
| **Affiliation** | **University of Glasgow** |
| **Date** | **18-May-2026** |
| **COI** | |

---

Apologies that I did not pick up on this during the first review of this article. I would appreciate you substituting 'lifestyle' for 'life circumstances' each time it is used. Words matter and there is a social justice reason for this framing. eg 'Homelessness is not a lifestyle choice'. I know this is not what is meant but the associative meaning is just too strong now! And framing this as circumstances is more accurate.

---

## VERSION 2 - AUTHOR RESPONSE

Reviewer: 2
Dr. Andrea Williamson, University of Glasgow
Comments to the Author:
Apologies that I did not pick up on this during the first review of this article. I would appreciate you substituting 'lifestyle' for 'life circumstances' each time it is used. Words matter and there is a social justice reason for this framing. eg 'Homelessness is not a lifestyle choice'. I know this is not what is meant but the associative meaning is just too strong now! And framing this as circumstances is more accurate.

*Thank you – we have adjusted the wording as advised.*

Reviewer: 2
Competing interests.: Not applicable.

---
